# STING-ing Pain: How Can Pro-inflammatory Signaling Attenuate Pain?

**DOI:** 10.1007/s12264-021-00672-1

**Published:** 2021-04-09

**Authors:** Wolfgang Liedtke

**Affiliations:** 1 Department of Neurology, Duke University School of Medicine, Durham, NC 27710 USA; 2 Department of Anesthesiology, Duke University School of Medicine, Durham, NC 27710 USA; 3 Department of Neurobiology, Duke University School of Medicine, Durham, NC 27710 USA; 4 Duke Neurology Clinics for Headache, Head-Pain and Trigeminal Sensory Disorders, Durham, NC 27705 USA; 5 Clinics for Innovative Pain Therapy, Department of Anesthesiology, Duke University, Raleigh, NC 27512 USA; 6 Chair of Neurology, Global Scientific Development Council, Regeneron Pharmaceuticals, Tarrytown, NY 10591 USA

Inflammation typically induces pain by producing pro-inflammatory mediators, but increasing evidence also indicates a role for inflammation in the resolution of pain by inducing anti-inflammatory and pro-resolution mediators [1, 2]. A recent *Nature* paper from Duke University, "STING controls nociception *via* type-I interferon signaling in sensory neurons", is noteworthy in this respect [3].

In this paper, Donnelly *et al*. from Ru-Rong Ji's lab report that activation of the STING (stimulator of interferon genes) signaling mechanism in nociceptive primary sensory neurons functions in an analgesic manner in naive and injured mice, using STING agonists, and applying sophisticated pain behavioral metrics to uninjured mice and mice with nerve constriction injury or a rigorous model of bone cancer pain. Their findings indicate that STING regulates steady-state nociception, which prompted the key discovery that analgesic STING signaling functions *via* type-I interferons (IFN-α or IFN-β). These findings, at the core of their new study, were derived from genetically-engineered mice, using behavioral and electrophysiological measurements from dorsal root ganglion nociceptor neurons. Particularly important mouse lines for these mechanistic studies were type-I interferon-receptor knockout mice, including cell-specific knockout in nociceptor sensory neurons. In these animals, behavioral evidence of analgesia in response to a type-I interferon and the neurophysiologic correlates of nociceptor action potential formation and calcium currents were missing, suggesting autocrine signaling in the dorsal root ganglion.

Notably, both pro-nociceptive and anti-nociceptive inflammatory mediators can contribute to the ensemble of inflammation. While type-II interferon (IFN-γ) is a typical pro-inflammatory cytokine, type-I interferons (IFN-α and IFN-ß) can be both pro-inflammatory and anti-inflammatory, depending on context. Previous studies have demonstrated anti-nociceptive actions of type-I interferons in the central nervous system [4]. Notably, STING is a strong inducer of type-I interferons in immune cells following infections or tissue injury [5, 6]. However, the role of STING in neurons, in particular primary sensory neurons, and in a larger context its role in pain have not been investigated.

The Donnelly *et al*. paper is a formidable piece of work because it elucidates a novel and unexpected mechanism of how interferon signaling is analgesic *via* STING. The new STING story from Dr. Ji's lab stings the dogma of pro-inflammatory signaling being pro-algesic. Beyond its impact as a fundamentally new insight, its potential for translation into new analgesic treatments has to be recognized, also opening up several new avenues for further mechanistic insights.

The paper lays out a compelling, frequently not fully appreciated background and rationale, namely that pain is an instinct that, *via* its sentinel function, encodes avoidance behaviors that protect the organism from potential danger, that neural encoding of pain preprograms the organism for future danger. In this vital system, as it subserves the physical integrity of the organism, primary nociceptor sensory neurons are centerfold because they function as neural integrators of the primary danger signal, modulated by inflammation, and coordinating unspecific defense and also more specific immune responses. So inflammation "increases the heat", sensitizes these nociceptors, and represents a form of neural injury.

But now we know that it is not that simple, with profound implications. STING signaling, as part of the early inflammatory response, triggered by bacterial or viral infection as well as tissue injury, functions as a potent anti-nociceptive. Gain-of-function and loss-of-function studies of STING as presented by Donnelly *et al*. are deep and convincing. Gain-of-function of STING shows significant analgesic effects in naïve mice as well as in preclinical models of chemotherapy-induced painful polyneuropathy (CIPN), peripheral nerve constriction injury, and bone cancer pain. These favorable properties beget more advantageous features, namely independence of STING activation and the resulting analgesia from reward circuitry and lack of effect of the opioid receptor blocker naloxone.

To complement, the new results on loss-of-function of STING, both genetically-encoded and chemically-mediated, paint a clear picture of lowered pain thresholds, indicating hyperalgesia and overall increased pain sensitivity.

Both approaches unambiguously indict the primary sensory neurons, in particular nociceptors, in the dorsal root ganglion as a key cellular site of action for the observed behavioral and electrophysiological effects on nociception.

The work is primarily new, unexpected, and relevant for basic science as well as translational medical science, in the interdisciplinary arena of pain research - as one expects for a *Nature* paper. However, sections of the paper are outright elegant in approach. What impresses are the rescue experiments of STING activation in STING pan-nulls *versus* STING conditional knockout (cKO) mice, whereby selective STING activators were completely inert in pan-null mice, but showed delayed rescue of pain hypersensitivity in the cKO animals. This clarifies the role of sensory neuronal STING as critical for nociceptor function in the early pain response. Delayed analgesic effects of STING activators were present in mice that did not express STING in nociceptor neurons but elsewhere, so that the cellular site for these effects is now open for discovery.

STING enhances the expression and secretion of type-I interferons (IFN-α and IFN-ß) by nociceptors which then signal in an autocrine/paracrine manner to interferon receptors, expressed by nociceptors. Importantly, this signaling critically involves TYK2 kinase, not PI3-kinase, and not MAP-kinases. TYK2 kinase signaling, in turn, then attenuates the pro-nociceptive ion channel function of voltage-gated sodium channels, amongst them Nav1.7, that are essential for the generation of action potentials [7], and voltage-gated calcium channels that are critical for neurotransmitter release. The forceful exclamation marks of this impressive study are partial validation of the new pro-inflammatory yet analgesic STING mechanism in human DRG neurons, and skillfully-conducted studies in macaques, by primate pain researcher Dr. Mei Chuan Ko at Wake Forest University.

**Fig. 1:**
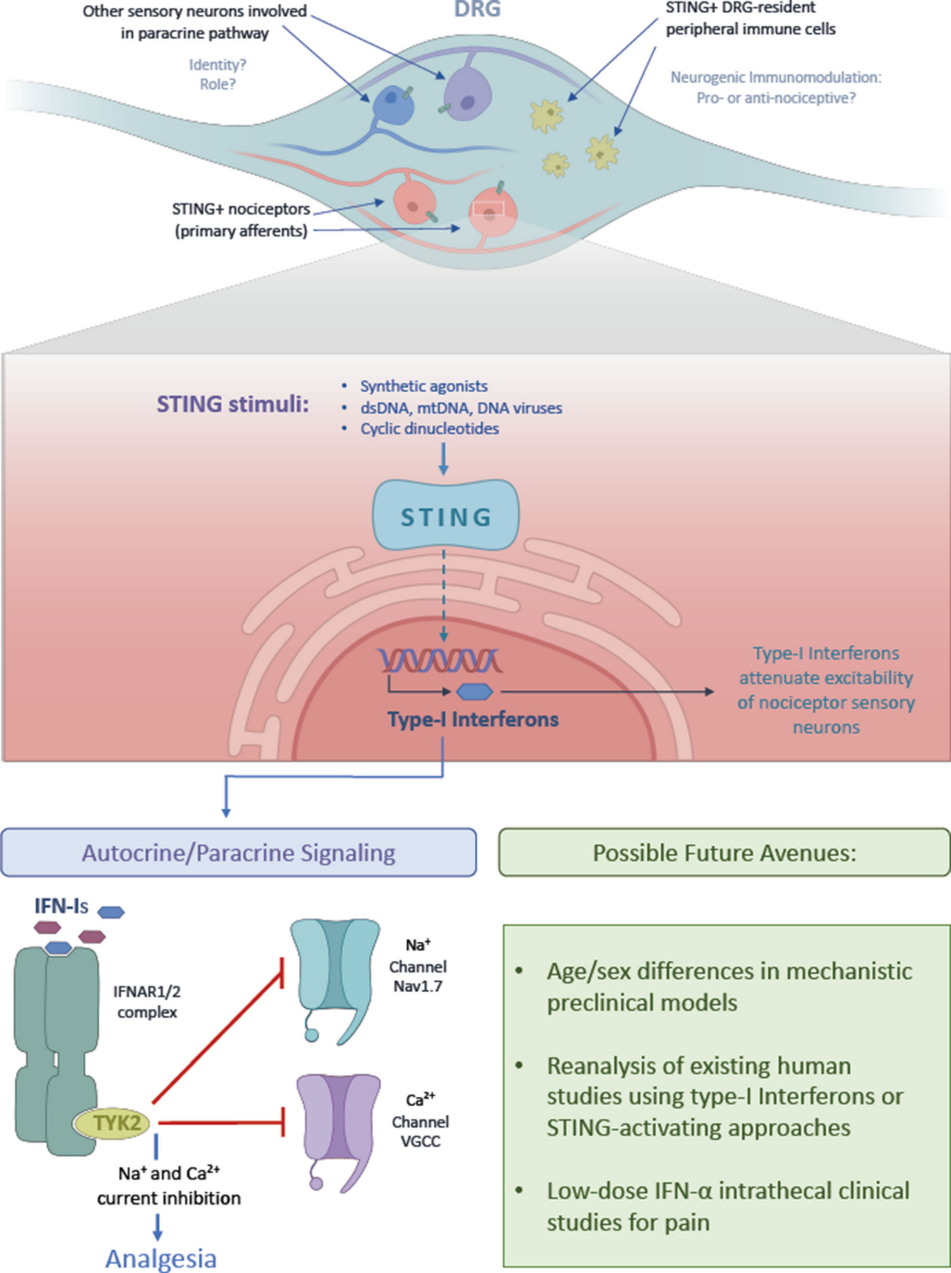
Summary of concepts of the new STING paper [3] and future directions.

As we look forward, this leaves us where? There appears to be a clear and direct translational opportunity, which is to use low-dose type-I interferons (IFN-α or IFN-ß, as used for adjuvant therapy of malignancy, in infections and autoimmune conditions) in chronic-refractory pain. One can consider the intrathecal route to avoid systemic effects, and apply low-dose interferon, perhaps the more widely used interferon-α, to patients with refractory CIPN or diabetic neuropathy pain. In addition, existing datasets and patients previously treated with low-dose IFN-α can be interrogated for whether this treatment led to diminished pain, in cases where there was pain before treatment. There appear to be 14 clinical studies referenced in clinicaltrials.gov. These trials were aiming for treatment of malignancies or chronic infections, not pain. In addition to trial application of IFN-α, STING agonists more recently used for adjuvant anti-malignancy treatments will be equally interesting, and whether treatment with them did diminish cancer/malignancy-associated pain (Fig. 1).

What might be a particularly interesting goal in the basic science arena is STING's effect on nociceptors, which will in turn influence immune cells. This begets the important question, how does feedback from immune cells modulate the analgesic effects of STING? In the case of immune-mediated attenuation of STING analgesic function, then STING's analgesic effects could be enhanced further by blocking (currently unknown) immune-mediated pro-algesic effects downstream of STING. Last but not least, the canonical "next" steps in the cookbook of pain research likely are already cooking: a more in-depth study of male/female sex differences [8], and how the new STING analgesic signaling plays out in young/old subjects?
